# Efficient chromatin profiling of H3K4me3 modification in cotton using CUT&Tag

**DOI:** 10.1186/s13007-020-00664-8

**Published:** 2020-08-31

**Authors:** Xiaoyuan Tao, Shouli Feng, Ting Zhao, Xueying Guan

**Affiliations:** College of Agriculture and Biotechnology, Zhejiang University, Hangzhou, 310058 China

**Keywords:** Cut&Tag, Cotton, Histone H3K4me3, ChIP

## Abstract

**Background:**

In 2019, Kaya-Okur et al. reported on the cleavage under targets and tagmentation (CUT&Tag) technology for efficient profiling of epigenetically modified DNA fragments. It was used mainly for cultured cell lines and was especially effective for small samples and single cells. This strategy generated high-resolution and low-background-noise chromatin profiling data for epigenomic analysis. CUT&Tag is well suited to be used in plant cells, especially in tissues from which small samples are taken, such as ovules, anthers, and fibers.

**Results:**

Here, we present a CUT&Tag protocol step by step using plant nuclei. In this protocol, we quantified the nuclei that can be used in each CUT&Tag reaction, and compared the efficiency of CUT&Tag with chromatin immunoprecipitation with sequencing (ChIP-seq) in the leaves of cotton. A general workflow for the bioinformatic analysis of CUT&Tag is also provided. Results indicated that, compared with ChIP-seq, the CUT&Tag procedure was faster and showed a higher-resolution, lower-background signal than did ChIP.

**Conclusion:**

A CUT&Tag protocol has been refined for plant cells using intact nuclei that have been isolated.

## Background

Epigenomic regulations of gene expression play key roles in the growth and development of multicellular organisms in which all cells harbor the same genomic sequences. Epigenomic regulations on the chromatic level, including DNA methylation, histone modification, and the differential binding of transcription factors and their recruited protein complexes, lead to differences in gene expression in different tissues and different developmental periods [1]. Chromatin immunoprecipitation (ChIP) with DNA sequencing is a widely applied chromatin profiling method for genome-wide mapping of DNA–protein interactions. However, the strategy suffers from its high background signal and false-positive artifacts caused by formaldehyde cross-linking and solubilization of chromatin during immunoprecipitation [2, 3]. Several alternative methods have been developed, including DNase1 footprinting [4], FAIRE-seq (formaldehyde-assisted isolation of regulatory elements sequencing) [5], Sono-seq (sonication of cross-linked chromatin sequencing) [6], MNase-seq (micrococcal nuclease sequencing) [7], and ATAC-seq (assay for transposase-accessible chromatin using sequencing) [8], to map transcription factor–binding sites. However, these profiling strategies are generally dependent on chromatin accessibility and cannot provide chromatin-binding information that is specific to any transcription factor. Enzyme-tethering strategies were developed to map chromatin protein-binding sites on intact cells and nuclei, including DamID (DNA adenine methyltransferase identification) [9], ChEC-seq (chromatin endogenous cleavage sequencing) [10], CUT&RUN (cleavage under targets and release using nuclease) [11], and CUT&Tag.

Similar to the DamID, ChEC-seq, and CUT&RUN strategies, CUT&Tag is an enzyme-tethering method in which the specific chromatin protein (e.g., histone, RNA polymerase II, or a transcription factor) is recognized by its specific antibody in situ, and it then tethers a Protein A (pA-Tn5) transposase fusion protein. The tethered pA-Tn5 transposase is activated by adding Mg^2+^. Because the pA-Tn5 fusion protein is already loaded with sequencing adapters, the generated fragments at chromatin protein-binding sites are integrated with adapters and ready for polymerase chain reaction (PCR) enrichment and DNA sequencing [3]. Compared with ChIP-seq, the CUT&Tag technology has more advantages, including (1) high resolution and a low background signal due to the activation of the transposase in situ to generate fragments; (2) freedom from the epitope masking caused by the cross-linking in ChIP; (3) a saving of time because the steps of the cross-linking of material and DNA sonication are not necessary; (4) integration of the fragments generated by the transposome with sequencing adapters, which are ready for PCR enrichment; and (5) a requirement for small amounts of starting material due to the procedure’s high sensitivity.

CUT&Tag was first designed for cultured mammalian cells. With the addition and binding of cells to concanavilin A–coated magnetic beads, CUT&Tag can be performed on a solid support [3]. Alternatively, the centrifuge method can be used to collect the cells or nuclei at low speed. The application of a similar enzyme-tethering strategy, CUT&RUN, was previously documented in *Arabidopsis* [12]. However, few CUT&Tag protocols were developed that were suitable for plants. Allotetraploid cotton is the largest natural fiber resource for textile products. The cotton genome is also a model for polyploid crop domestication and transgenic improvement because of its high-quality sequenced genomes [13, 14]. Here we use cotton as the model system for developing an effective CUT&Tag protocol for epigenomic research. We aimed to (1) set up the detailed steps for CUT&Tag that can be widely used in other plants; (2) compare the signal resolution of CUT&Tag with that of ChIP using the same starting material; and (3) provide the workflow and general information about required reads for polyploid plants to meet the efficient resolution required for bioinformatic analysis.

## Results

### Workflow of CUT&Tag-seq vs. ChIP-seq

**Fig. 1:**
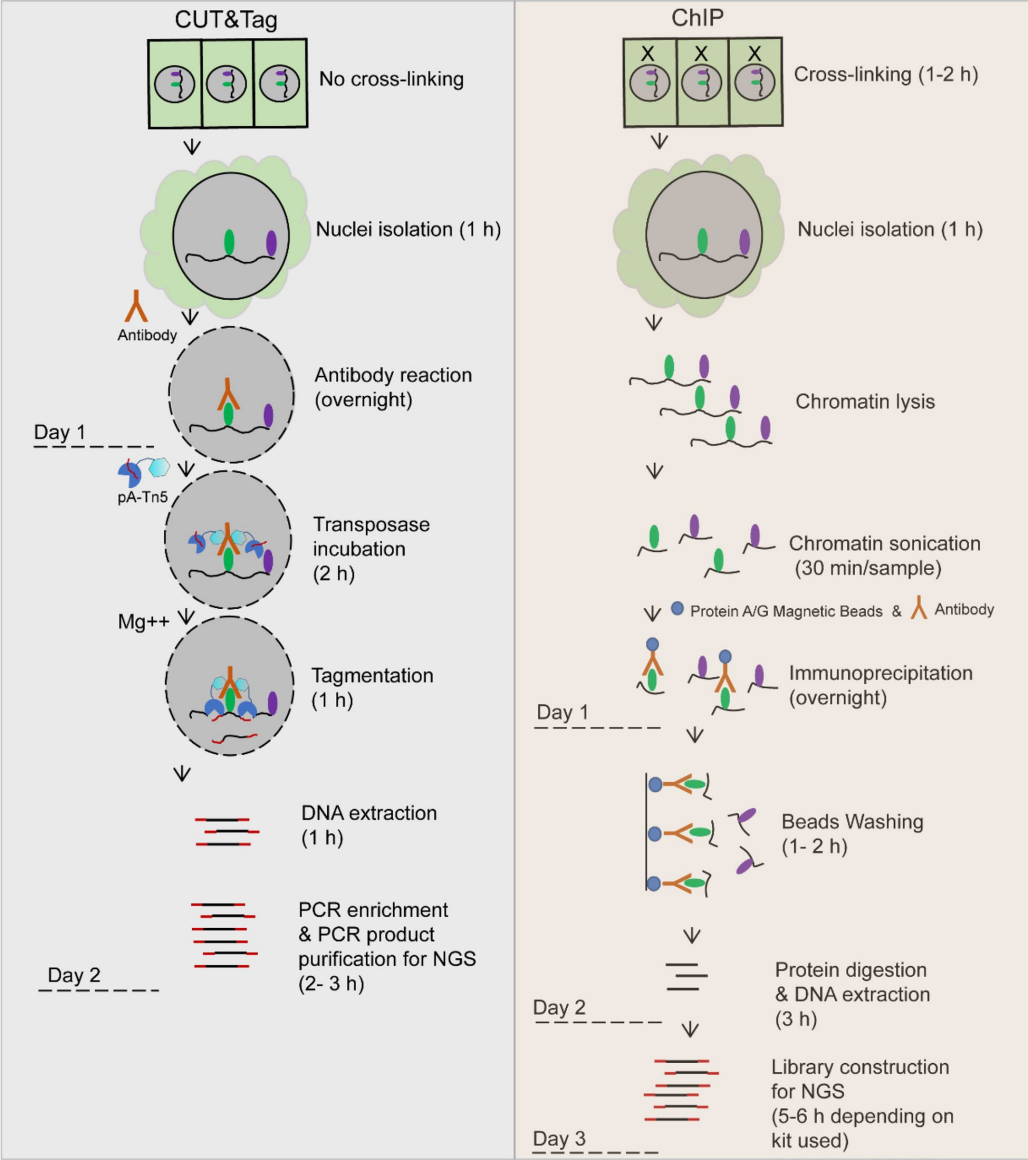
The workflow of CUT&Tag vs. ChIP. The performance time for each step was estimated roughly

The workflow of CUT&Tag and ChIP in parallel with the performing time for each step was roughly estimated (Fig. 1). The detailed method was described in the Materials and Methods section. Unlike ChIP, the CUT&Tag was applied with an in situ strategy, so no cross-linking was needed to stabilize the protein–protein and protein–DNA interactions. We found that cross-linking relied on formaldehyde in ChIP usually caused difficulties in isolating the nuclei with 20% Triton. In CUT&Tag, the intact nuclei were subjected to antibody incubation in the presence of a nonionic detergent, digitonin, which has been successfully used in other in situ methods [8, 10]. This allowed antibody permeabilization of the nuclei without compromising nuclear integrity. In the ChIP procedure, the chromatin lysis from the isolated nuclei needed to be sonicated into random fragments at 100–500 bp before the immunoprecipitation reaction with the antibody. We used a Bioruptor™ (Diagenode, Denville, NJ, USA) to shear the DNA (aliquot of 350 μL in each tube for sonication) to 100–500 bp in length. It usually takes at least 30 min for each sample. If the sample number increases, hours are needed in the sonication step. After the CUT&Tag or ChIP reaction, the DNA was isolated for library construction and NGS. As in ChIP, the DNA–protein was cross-linked; it was difficult to extract the DNA without reverse cross-linking. Alternatively, the protein can be digested with proteinase K before DNA extraction, which makes the performance time of the DNA isolation step longer compared with the CUT&Tag procedure. Finally, after the fragmentation of protein-binding chromatin by Tn5, the fragments were already integrated with adapters and ready for PCR enrichment and NGS. In comparison, it took 4–5 h longer to construct the NGS library for the ChIP DNA we obtained. In summary, the CUT&Tag procedure outperforms the ChIP procedure in operational simplicity and experimental time needed.

### Nuclei used in CUT&Tag can be semi-quantified by DNA determination

**Fig. 2:**
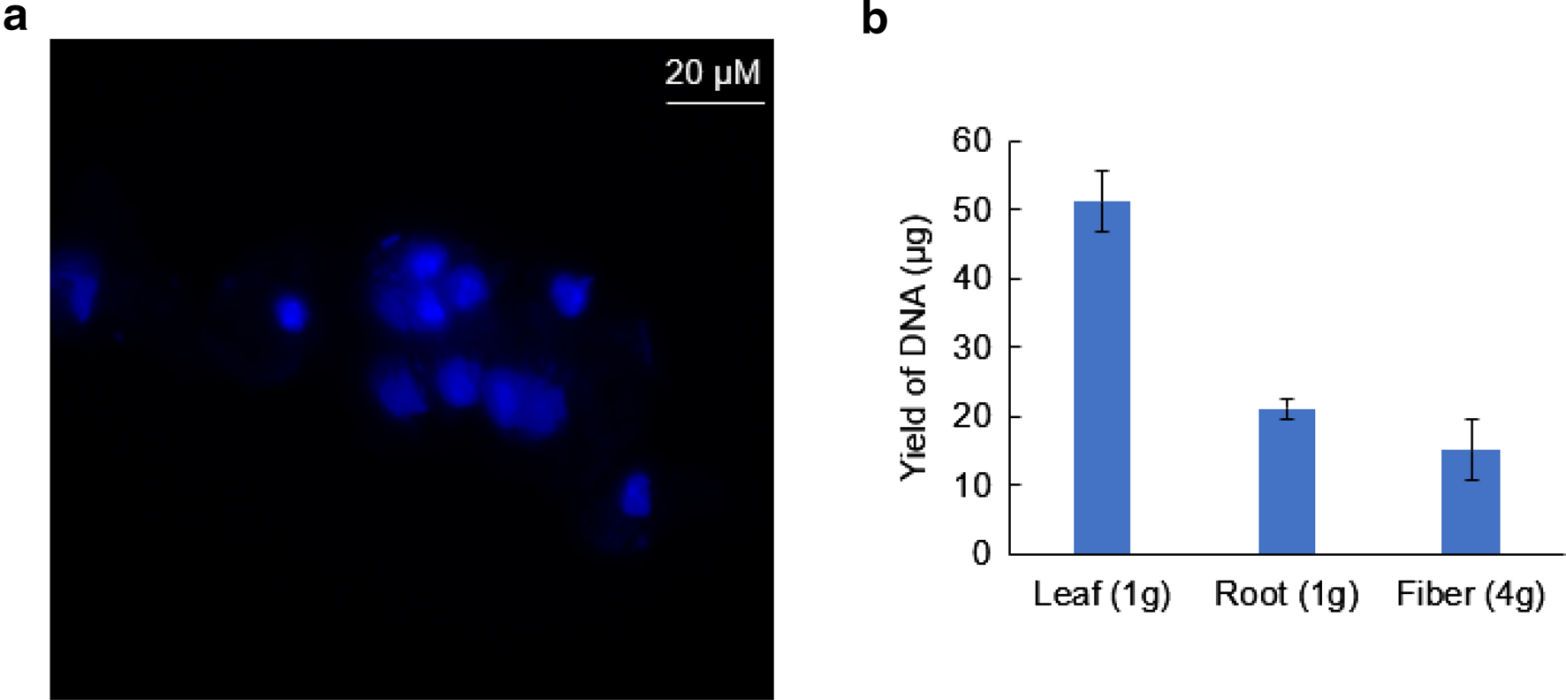
Determination of the amount of starting nuclei in the CUT&Tag reaction by DNA quantification. **a** DAPI staining of intact nuclei. **b** Quantification of DNA extracted from different tissues as indicated. Cotton (*G. barbadense*, accession H7124) leaf and root from 4-week-old seedlings were used. Fibers from three or four cotton balls (20 D cotton fiber) of H7124 were used

The presence of the cell wall in plant cells makes it difficult for antibody to penetrate the cells. As an alternative, intact nuclei were used in the assay (Fig. 2a). The other unknown was the amount of nuclei that should be used in each CUT&Tag reaction. We found it was difficult to count the number of nuclei under the microscope because the nuclei isolated from plants usually clustered together. We tried to semi-quantify the nuclei by determining the DNA that could be extracted. In the test for histone H3K4me3 modification in the leaves of cotton (*G. barbedense*, accession H7124), 150 µL of nuclei suspension was used in each CUT&Tag reaction (step 9 in the protocol), which equal to ⁓ 1.5 µg of chromatin according to the semi-quantification of nuclei by DNA determination (Fig. 2b). We also semi-quantified the nuclei isolated from different tissues including root and fiber of cotton (*G. barbedense*, accession H7124), results indicated that nuclei from 1 g root or 4 g fiber (from 3–4 20 D cotton balls of H7124) equal to 15–20 µg of chromatin, which was enough for 10 CUT&Tag reactions.

### CUT&Tag biological replicates showed high repetitiveness and high signal-to-noise ratio

**Fig. 3:**
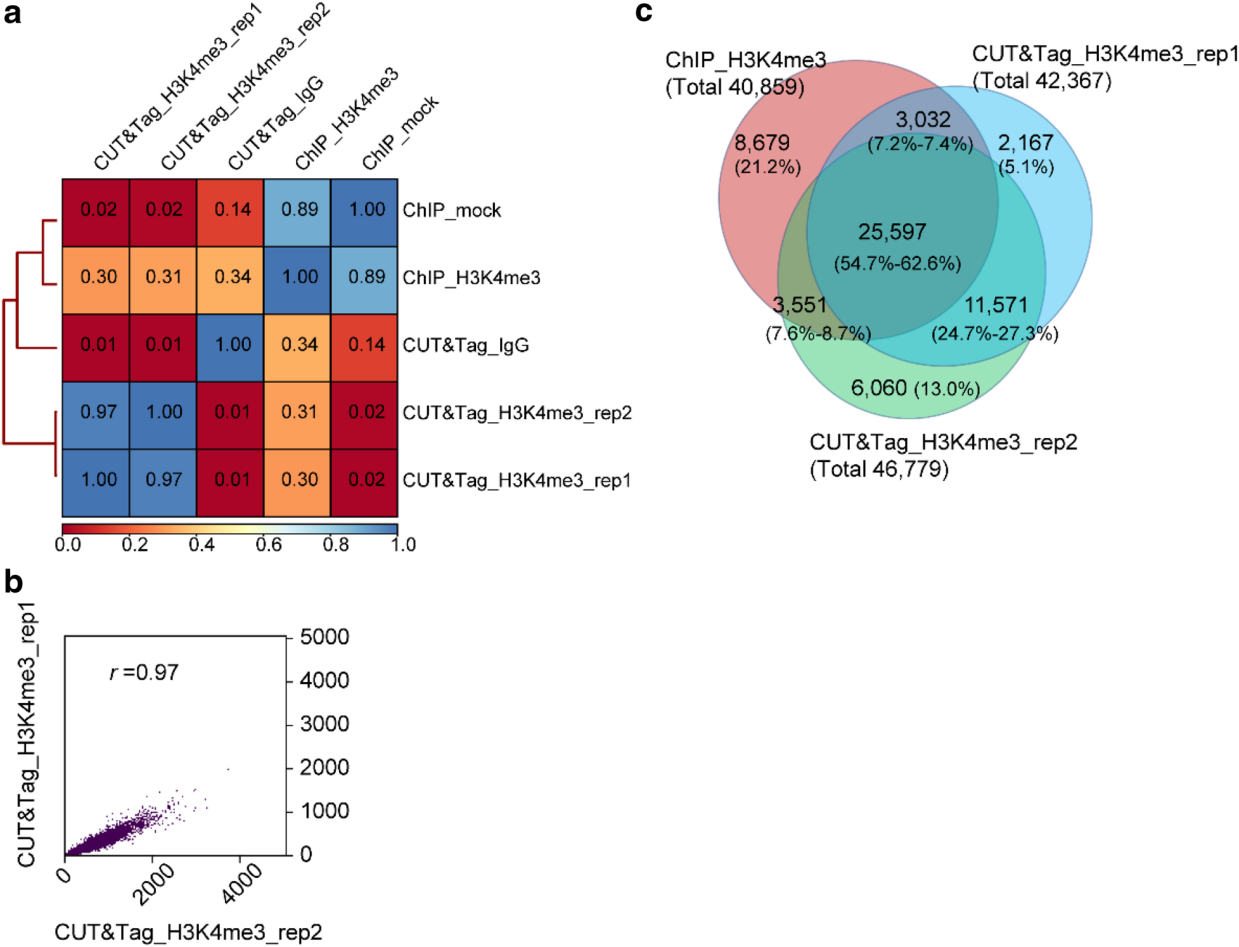
Correlation analysis of CUT&Tag and ChIP samples. **a** Hierarchically clustered correlation matrix of CUT&Tag replicates (rep1 and rep2) and with ChIP-seq profiling for the H3K4me3 histone modification. The same antibody was used in all experiments. Pearson correlations were calculated in deepTools (the multiBamSummary was followed with plotCorrelation tools) using the read counts split into 500-bp bins across the genome. **b** Scatterplot correlation of CUT&Tag replicates (rep1 and rep2). Pearson’s *r* was indicated. (**c**) Number of shared peaks and unique peaks in CUT&Tag replicates (rep1 and rep2) and ChIP-seq. Peaks were called by macs2 using randomly sampled 6-M clean data of CUT&Tag and 24-M clean data of ChIP. Peaks overlapped across the genome and with the distance of peak summit < 300 bp were considered as the same peak

Trimethylation of lysine 4 of histone H3 (H3K4me3) is a universal active marker of gene expression. We set up two biological replicates for the CUT&Tag reaction of H3K4me3 antibody. The reaction of each replicate was set up separately at the beginning using the intact nuclei isolated. The CUT&Tag reaction with IgG antibody was used as a control. The ChIP for H3K4me3 antibody was set up using the same material, and the ChIP mock reaction without the addition of H3K4me3 antibody was used as a control. Qubit analysis was performed after PCR enrichment and purification. Results indicated that the CUT&Tag_IgG control showed a low background signal, and the two replicates of the CUT&Tag_H3K4me3 group had fragments with a peak size of ~ 350 bp (Additional file 1: Figure S1), indicating the successful fragmentation of the chromatin. We then performed NGS and mapped the clean reads to the reference genome [14]. We obtained 10.61 million (M), 15.30, and 14.16 M mapped reads for two replicates of CUT&Tag profiling for H3K4me3 and the IgG control, respectively (Table 1). In comparison, we carried out parallel H3K4me3 profiling using the conventional ChIP procedure. The NGS generated mapped reads of 23.17 and 31.34 M for ChIP and its mock control, respectively (Table 1). We first did the correlation analysis for the CUT&Tag and ChIP groups, and results indicated that both of the replicates of CUT&Tag showed a very low correlation with the CUT&Tag_IgG control (*r* = 0.01, Pearson’s correlation), indicating that the CUT&Tag experimental group and the control group varied significantly and the CUT&Tag experimental group was different from the background noise (Fig. 3a). In comparison, the ChIP_H3K4me3 group showed a high correlation with its mock control (*r* = 0.89, Pearson’s correlation), which indicated that the signal-to-noise ratio in the ChIP assay would become a problem (Fig. 3a). We also dot plotted the correlation of the two replicates of CUT&Tag_H3K4me3. Data showed that they had a near perfect correlation (*r* = 0.97, Pearson’s correlation) (Fig. 3b), indicating the high repetitiveness within different biological replicates.

**Table 1:** NGS data summary of CUT&Tag-seq

Sample name	Raw base (G)	Clean base (G)	Raw reads	Clean reads	Mapped reads (% of clean reads)	Unique mapped reads (% of mapped reads)	Unique deduplicated reads (% of unique mapped reads)
CUT&Tag_H3K4me3_rep1	3.39	3.30	11,301,203	11,014,206	10,613,503 (96%)	10,405,490 (98%)	8,358,016 (80%)
CUT&Tag_H3K4me3_rep2	7.00	4.70	23,331,123	15,659,491	15,297,385 (98%)	14,779,742 (97%)	10,706,214 (72%)
CUT&Tag_IgG	7.26	4.81	24,191,937	16,044,295	14,160,416 (88%)	9,666,045 (68%)	770,447 (8%)
ChIP_H3K4me3	7.66	7.60	25,526,344	25,343,026	23,173,732 (91%)	22,052,284 (95%)	12,604,105 (57%)
ChIP_mock	9.99	9.92	33,289,788	33,071,131	31,341,880 (95%)	28,311,798 (90%)	16,363,867 (58%)

In order to evaluate the signal resolution between the CUT&Tag and ChIP data, we randomly sampled the same depth of sequencing reads ranging from 6 to 24 M from each sample and summarized the number of called peaks from them. Results showed that 42,367 and 46,779 peaks were called from two replicates of CUT&Tag, respectively, but only 18,024 peaks were called from the ChIP data when using 6-M clean reads (Table 2). There were 40,859 peaks called when using as much as 24-M clean reads from ChIP (Table 2), which means that 6-M clean reads of CUT&Tag can provide signals equivalent to 24-M clean reads of ChIP. The The overlapped peaks were determined using the peaks from 6-M clean data of the CUT&Tag and 24-M clean data of the ChIP. Among these peaks, 25,597 (54.7–62.6%) peaks were shared by CUT&Tag and ChIP, and 37,168 (79.5–87.7%) peaks were shared between two replicates of CUT&Tag, indicating the high reproducibility of two replicates of CUT&Tag data (Fig. 3c). The FRiP (fraction of reads in peaks) values calculated the ratio of mapped reads that fall into peaks among all mapped reads, and they act as indicators of the signal-to-noise ratio [15]. The FRiP value for each group of peaks was calculated, and the results indicate that CUT&Tag generated high signal-to-noise ratio (FRiP = 0.7; Table 2). These results suggest that CUT&Tag has higher signal resolution compared with ChIP.

**Table 2:** Number of called peaks and FRiP value under the same sequencing depth as indicated

Sample	6 M	8 M	10 M	12 M	14 M	16 M	24 M
CUT&Tag_H3K4me3_rep1	42,367 (0.70)	45,417 (0.72)	47,189 (0.74)	–	–	–	–
CUT&Tag_H3K4me3_rep2	46,779 (0.66)	49,775 (0.68)	53,140 (0.70)	55,667 (0.71)	56,331 (0.72)	–	–
CUT&Tag_IgG	1,082 (0.44)	1,119 (0.44)	1,135 (0.44)	1,159 (0.44)	1,187 (0.44)	–	–
ChIP_H3K4me3	18,024 (0.11)	23,411 (0.13)	27,491 (0.15)	30,898 (0.16)	33,543 (0.17)	35,495 (0.18)	40,859 (0.20)
ChIP_mock	602 (0.01)	843 (0.01)	1,029 (0.01)	1,317 (0.01)	1,682 (0.01)	–	–

Data were generated by random sampling of clean reads from the NGS fastq files. FRiP (Fraction of reads in peaks) [15] values which act as an indicator of a signal-to-noise ratio were provided within the brackets.

**Fig. 4:**
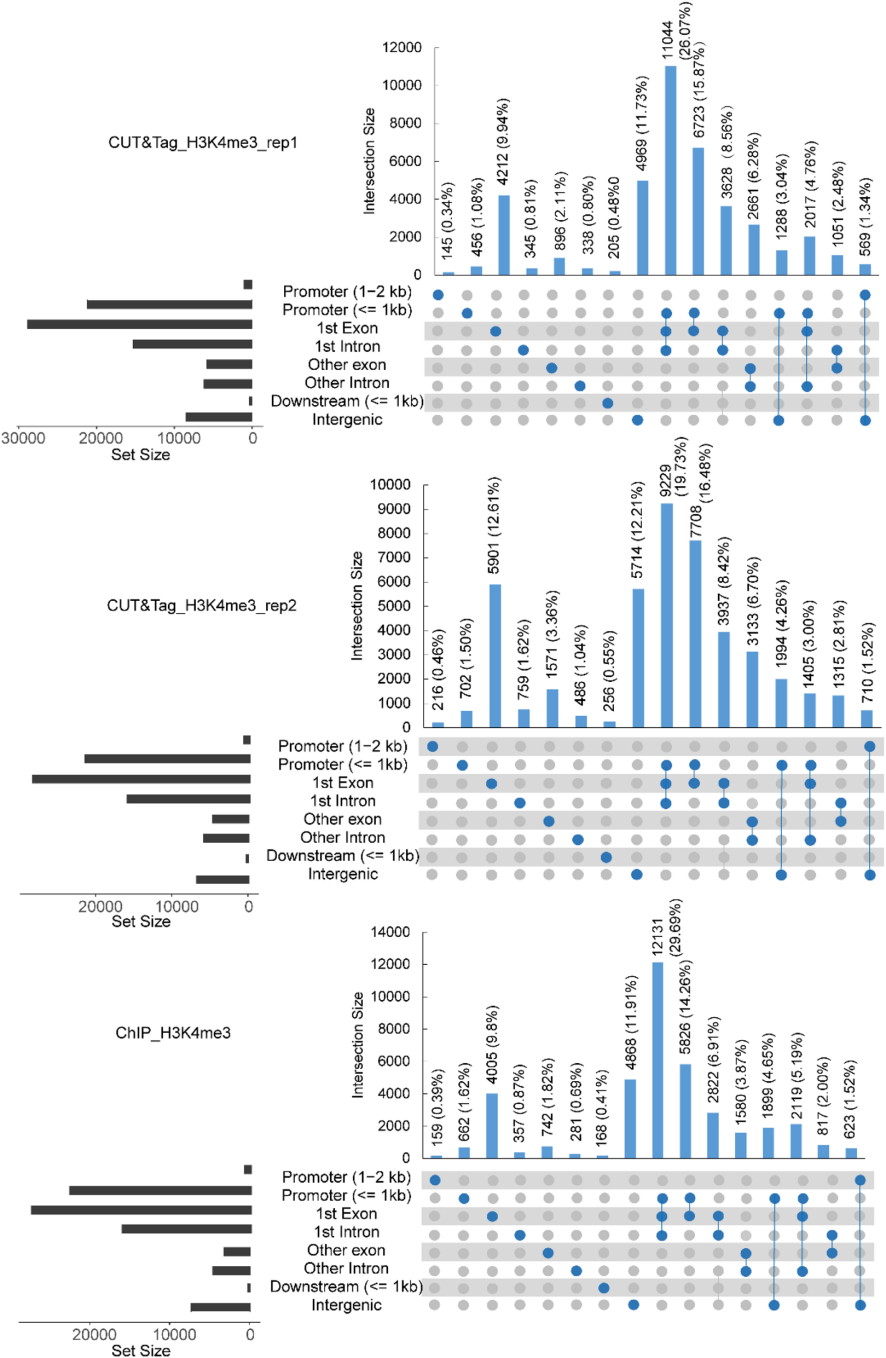
The histogram diagram showed the annotation of peaks for the H3K4me3 histone modification from CUT&Tag and ChIP data. **a**, **b** Peak distribution in CUT&Tag replicates (rep1 and rep2). **c** Peak distribution in ChIP. Peaks were called by macs2 using randomly sampled 6-M clean data of CUT&Tag and 24-M clean data of ChIP

**Fig. 5:**
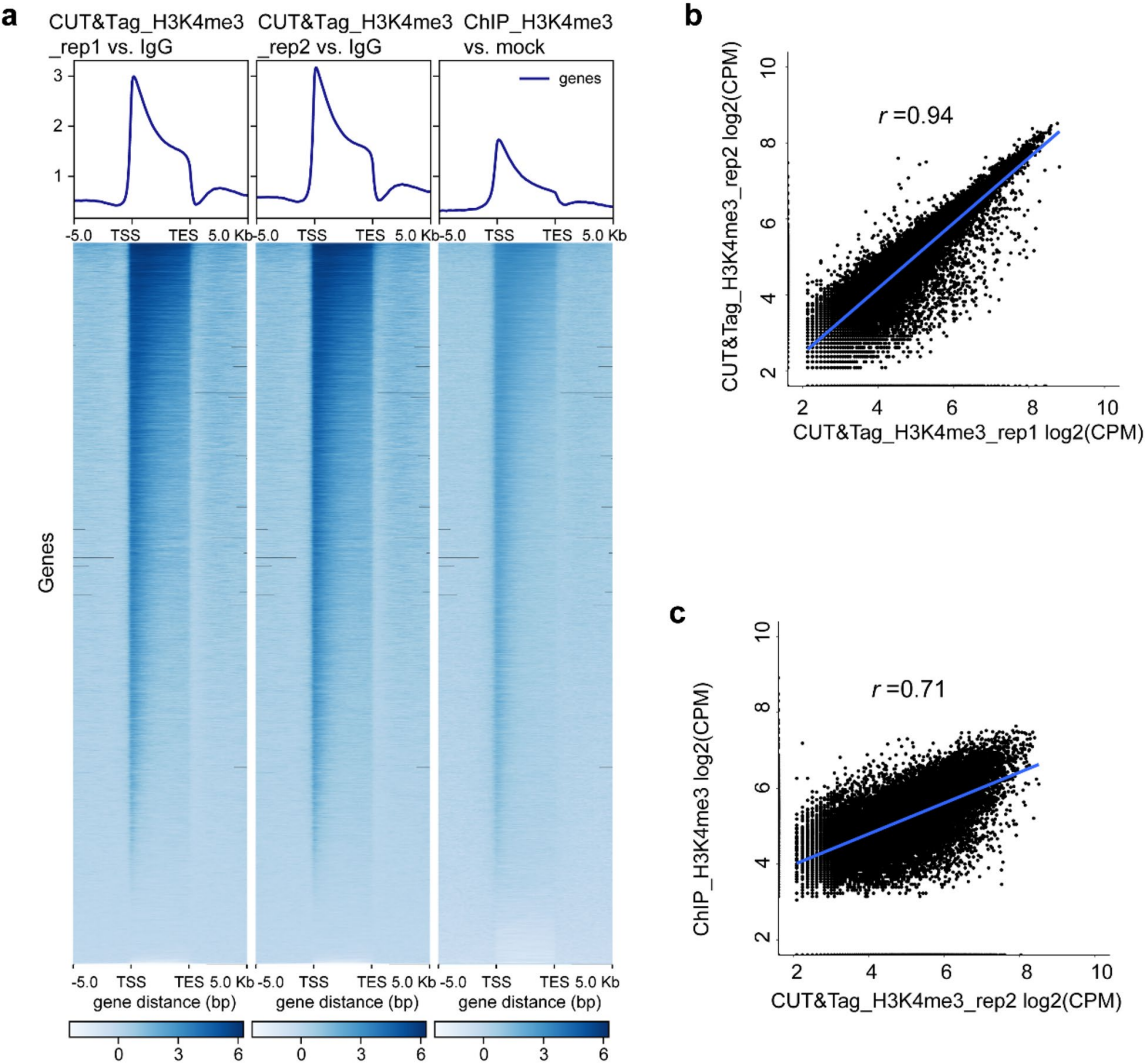
Analysis of H3K4me3 signals near the protein coding genes in CUT&Tag and ChIP. **a** Heatmap of CUT&Tag and ChIP signals upstream and downstream of the gene body. Scale regions were 5,000 bp upstream of the translation starting site (TSS), 5,000 bp downstream of the translation end site (TES), and a 5,000-bp region on the gene body. Length was plotted using computeMatrix and plotHeatmap tools in deepTools. **b**, **c** The dot plots showed the correlation analysis of peaks near the genes. Signals of the peaks were normalized by the log_2_ value of count per million mapped reads (CPM)

The genomic locations of the peaks were divided into eight categories, including 1–2 kb promoter (1–2 kb 5′ upstream of translation starting site), 1-kb promoter (≤ 1-kb 5′ upstream of translation starting site), first exon, first intron, other exon, other intron, 1-kb downstream (≤ 1-kb 3′ upstream of translation terminating site), and intergenic (out of the region described above). Here we only summarized the distribution of peaks called using 6-M clean reads of CUT&Tag-seq data and 24-M clean reads of ChIP-seq data. The H3K4me3 signals from both the CUT&Tag and ChIP data were predominantly (60–70%) enriched in the 1-kb promoter, first exon, and first intron categories (Fig. 4). This is consistent with previous reports showing that H3K4me3 signals were mainly located in the promoter and 5′ regions of the gene [16, 17]. However, on the heatmap of all of the H3K4me3 signals normalized with the CUT&Tag_IgG control or ChIP_mock control in the region of the gene body and its 5-kb flanking region, the signals from CUT&Tag had higher intensities than those from ChIP-seq (Fig. 5a). The correlation analysis of peaks near the genes showed a high correlation between two CUT&Tag replicates (Fig. 5b, *r* = 0.94, Pearson’s correlation), and a strong correlation between CUT&Tag and ChIP (Fig. 5c, *r* = 0.71, Pearson’s correlation).

**Fig. 6:**
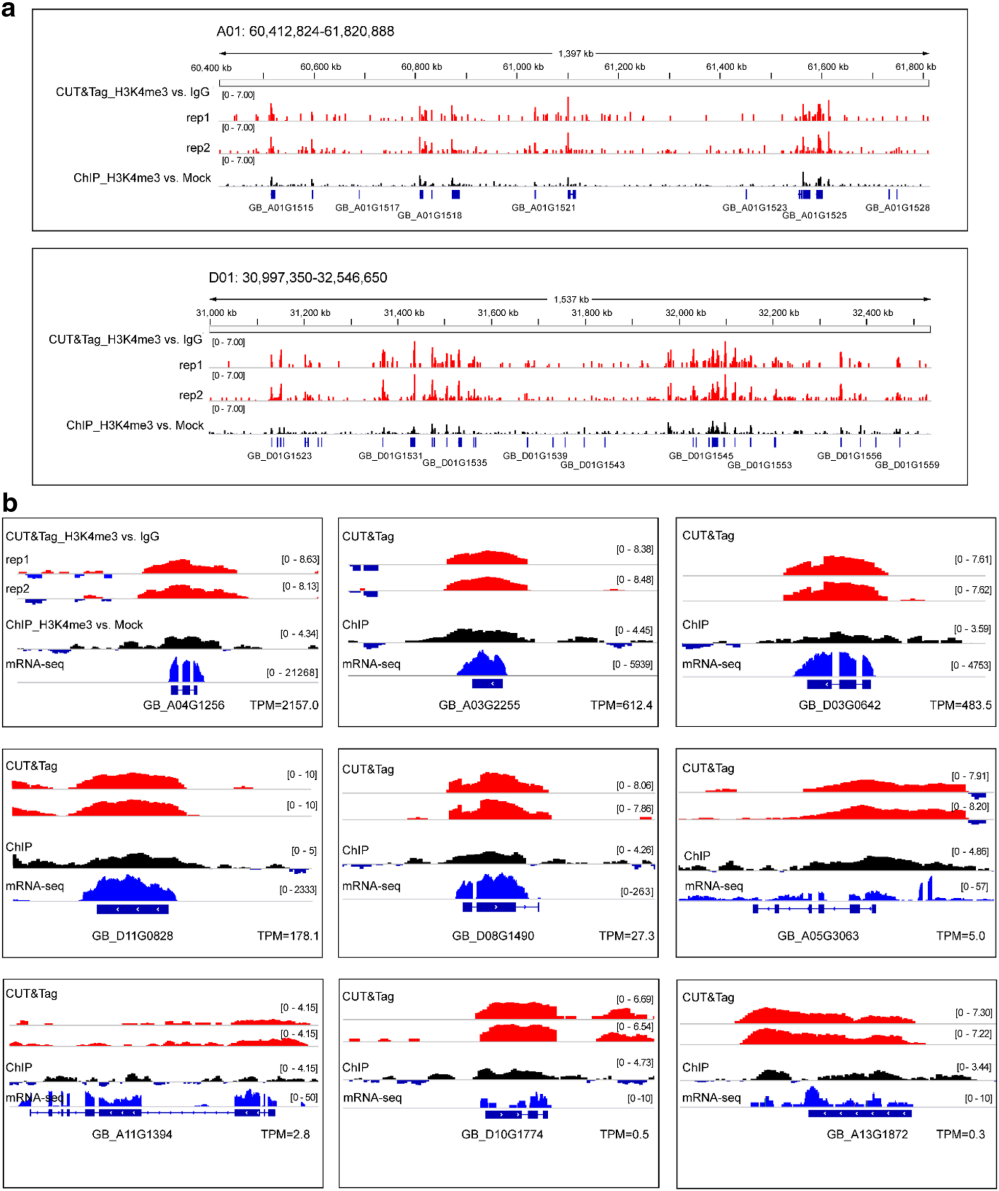
Representative IGV screenshot for H3K4me3 signals. **a** Representative IGV overview of CUT&Tag signals compared with those of the ChIP assay across a large genome region. ~ 1600 kb genome regions were randomly selected. **b** Representative IGV screenshot for genes with varied expression levels showed high resolution of CUT&Tag signals compared with those of the ChIP assay. The normalized bigWig files generated from bamCompare by comparing the treatment bam file (CUT&Tag or ChIP reaction) and the control bam file (IgG or mock control) were used. TPM, transcripts per kilobase of exon model per million mapped reads

As an additional step, we observed the H3K4me3 signals of both a large genome region (i.e., a randomly selected region covering ⁓ 1600 kb) and a small chromatin region of individual genes (selected with different expression levels) in the CUT&Tag and ChIP data using Integrative Genomics Viewer (IGV) software [18]. Consistent with the heatmap intensities indicated, the CUT&Tag signal outperformed the ChIP signal in resolution and sensitivity (Fig. 6a), especially in those genes with relatively low expression (e.g., the genes in GB_A11G1394, GB_D10G1774, and GB_A13G1872 in Fig. 6b). Overall, the CUT&Tag signal showed higher resolution and lower background noise for H3K4me3 profiling genome-wide.

### Histone H3K4me3 signal intensities are associated with active gene expression

**Fig. 7:**
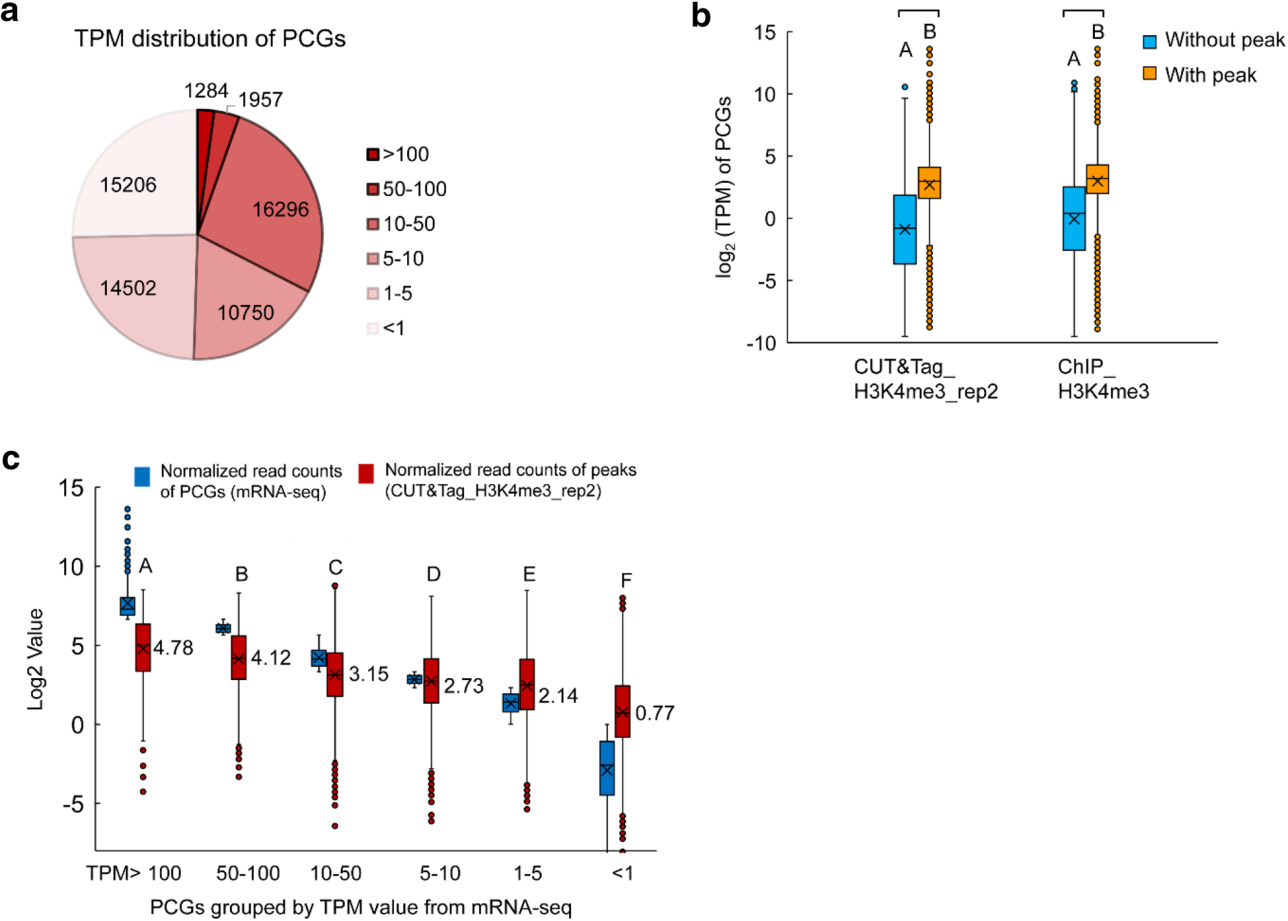
Histone H3K4me3 signal intensities are associated with active gene expression.** a** TPM distribution of PCGs. **b** Box plot showing normalized transcript levels (log_2_TPM), determined by mRNA-seq, at two subclasses of genes that with or without H3K4me3 peaks in CUT&Tag-seq or ChIP-seq. Mean values of A and B indicate significant different between two subclasses (*P* < 0.001, Fisher Pairwise Comparisons). **c** Box plot showing normalized H3K4me3 peak signals (log_2_TPM), determined by CUT&Tag-seq, at a number of different subclasses of genes that ranged and artificially divided by TPM values from mRNA-seq. Blue box: Normalized read counts of PCGs from mRNA-seq by log_2_TPM. Red box: Normalized read counts of peaks from CUT&Tag-seq by log_2_CPM. Mean values of log_2_CPM of the H3K4me3 peak signals were indicated. Values that do not shared a letter (from A to F) are significant different (*P* < 0.001, Fisher Pairwise Comparisons)

The allotetraploid cotton *G. barbadense* harbors a genome of approximately 2.22 Gb in size, with 75,071 high-confidence protein-coding genes (PCGs) [14]. We did the transcriptome sequencing for the same leaf issue and identified 44,789 genes expressed with a TPM (transcripts per kilobase of exon model per million mapped reads) that greater than 1 (Fig. 7a). We further examined the number of peak-related PCGs (with peaks located within genes and a flanking region of ≤ 1 kb). Results showed that there were 38,513 and 42,265 peak-related PCGs from two replicates of CUT&Tag-seq, respectively, which covered 34,072 (76.1%) and 36,988 (82.6%) of the expressed genes with a TPM of greater than 1. In comparison, 33,229 peak-related PCGs from ChIP-seq covered 30,016 (67.0%) of the expressed genes with a TPM of greater than 1. Thus, H3K4me3 modification is a nearly universal histone modification that is well documented to be associated with the active transcription of genes [16, 19–21]. The correlation analysis between the intensities of gene-associated H3K4me3 signals and the transcriptional levels of corresponding genes was performed. Results indicated that the H3K4me3 intensities of gene related peaks had a weak correlation with gene expression levels (Additional file 1: Figure S2, *r* = 0.31). However, a descending trend of H3K4me3 signals in the heatmap was found when the plotted genes were arranged in the descending order of their TPM (Additional file 1: Figure S3). Instead, we further boxplot the expression levels of genes that divided into two different subclasses of with or without CUT&Tag-seq peaks, results showed that PCGs with H3K4me3 peaks are significantly higher expressed (Fisher Pairwise Comparisons, *P* < 0.001) (Fig. 7b). Alternatively, we boxplot the H3K4me3 peak intensities from CUT&Tag-seq at six different subclasses of genes that descending ordered and artificially divided by TPM values from mRNA-seq (TPM > 100, 50–100, 10–50, 5–10,1–5 and < 1), results showed that the corresponding H3K4me3 signal intensities in each group of genes decreased significantly (Fisher Pairwise Comparisons, *P* < 0.001) (Fig. 7c). These data indicated that histone H3K4me3 signal intensities are associated with active gene expression.

## Discussion

Plant tissues is still very challenging due to the presence of the cell walls, large vacuoles, and secondary metabolites [22]. The isolation of plant chromatin needs a plant-specific approach; for example, nuclei of high quality need to be isolated before chromatin lysis is performed [22]. Cotton fiber is a specialized cellulosic tissue from which it is difficult to isolate enough nuclei for a ChIP reaction. Slight modification in the procedures of nuclei isolation and PCR enrichment after fragmentation is recommended if the amount of starting material is small at the signal cell level, such as anthers, fibers, and ovules. We highly recommend optimizing the Triton incubation time for nuclei isolation. The nuclei in CUT&Tag must be intact. Broken nuclei will lead to the non-specific tethering of Protein A (pA-Tn5) transposase fusion protein to the chromatin, subsequently the non-specific fragmentation in situ arises a high level of background noises in CUT&Tag. In the original study [3], the addition and binding of cells to Concanavalin A–coated magnetic beads was performed, allowing magnetic handling of the intact cells in all successive washing and reagent incubation steps. This step can be replaced by gentle centrifugation (< 600 × *g*) between steps [3]. Our data showed that gentle centrifugation (300 × *g*) to precipitate the nuclei works well. For antibody efficiency, H3K4me3 is an abundant chromatin modification mark that can generate sufficient signals for profiling. For other chromatin modification marks or chromatic proteins with relatively low abundance, a secondary antibody against the protein-specific primary antibody is recommended to amplify the signal [3]. Because the antibody binds to the epitopes in situ and CUT&Tag has high sensitivity, antibodies successfully tested in immunofluorescence would work with CUT&Tag. Accordingly, CUT&Tag in transgenic plants tagged with a GFP or His fused target protein can be used with the anti-tag antibody instead of the protein-specific antibody.

Regarding the NGS depth for CUT&Tag, it was reported that approximately 8 M mapped reads of the human genome (~ 3 Gbp in size) displayed a clear pattern for lysine-27-trimethylation of the histone H3 tail (H3K27me3), an abundant histone modification that marks silenced chromatin regions [3]. In addition, CUT&Tag populated peaks at low sequencing depths, where approximately 2-M reads are equivalent to 8-M reads for CUT&RUN (or 20 M for ChIP-seq), demonstrating the exceptionally high efficiency of CUT&Tag [3]. It was documented that 6- to 8-M unique deduplicated reads by CUT&RUN could provide genome-wide H3K27me3 landscapes with high sensitivity, specificity and reproducibility in the model plants of *Arabidopsis* harbors a genome of 125 Mbp in size that encoded 25,498 PCGs [12]. According to our data, 6- to 8-M clean reads from CUT&Tag are equivalent to 24-M clean reads for ChIP-seq (Table 2); and 8-M unique deduplicated reads from CUT&Tag is sufficient for profiling the H3K4me3 signal genome-wide for allotetraploid cotton plants with the genome size of approximately 2.2–2.3 Gbp which encoded ~ 75,000 high-confidence PCGs. Regarding the cost of sequencing and differences in plant genome size and the number of PCGs, pilot sequencing is recommended for your libraries (e.g. sequencing 2–3 G raw base using 150 × 150 bp paired-end sequencing) first to test the sensitivity of CUT&Tag libraries in your plants, and then did more sequencing if needed.

Based on findings in the previous publication [3], the Pearson’s correlation coefficient *r* value between CUT&Tag and ChIP profiling for the H3K4me1 histone modification is 0.7–0.8. We did the same correlation analysis using the same parameter and found that the Pearson’s correlation coefficient *r* value is 0.3 between CUT&Tag and ChIP for H3K4me3 (Fig. 3). The low *r* value is mainly caused by the different profiling procedures of the methods (i.e., fixed chromatin in ChIP vs. native chromatin in CUT&Tag; fragmentation of the DNA by sonication to ~ 500 to 1000 bp in size in ChIP vs. fragmentation of the DNA in situ by Tn5 transposase to ~ 350 bp in size); this leads to heterogeneity between CUT&Tag and ChIP. However, when we perform dot plotting of the correlation of peaks signals near the genes, the *r* value is 0.71 between CUT&Tag and ChIP (Fig. 5c), the peaks signals generated from both of the methods showed high homogeneity. Also, the Pearson’s correlation coefficient *r* value between ChIP-seq and its mock control is high, indicating low signal-to-noise ratio in ChIP assay. For this reason we are seeking a more efficient chromatin profiling method for our research on epigenetics in cotton. In our study so far we have successfully established a CUT&Tag protocol for cotton that can also be widely applied to other plants.

Cotton plants (*Gossypium* spp.) bear seed trichomes (cotton fibers) that are an important commodity worldwide. Until now, the profiling of epigenomic modifications in cotton fibers was difficult because of the amount of starting materials required to harvest enough chromatin. Cotton fibers are single-cell structures. After differentiation, the fiber cells move into a stage of rapid elongation to increase the cell length up to 2–3 cm without cell division. This means the nuclei do not increase during the fiber cell-elongation stage. The chromatin enrichment for fiber in the elongation stage requires large amounts of fiber tissue at relatively low efficiency. We are interested in the nuclei that can be isolated from cotton fibers. From the DNA extracted, we found that fiber nuclei extracted from four cotton balls (20 D cotton fiber) were sufficient for about 20 CUT&Tag reactions (Fig. 2b). In comparison, according to our experience, at least 20 µg of chromatin is needed in each ChIP reaction to obtain enough DNA for the library construction of cotton. Thus the CUT&Tag needed only approximately 1/20 of the starting material needed by the conventional ChIP strategy. In addition, few chromatin profile methods were successfully applied to study the specific transcription factors that play key roles in regulating fiber differentiation and elongation. The CUT&Tag we established provided a promising strategy for further application in the study of epigenomics in cotton fiber development.

Histone modification that alters the nucleosome structure and recruits regulatory proteins is recognized as an integral part of the gene regulation in eukaryotes from yeasts to humans. The trimethylation of lysine 4 of histone H3 (H3K4me3) is one of the most established histone modifications. It has a well-established association with gene expression [23], is often described as an “activating” histone modification, and is assumed to have an instructive role in the transcription of genes. However, it has not been convincingly supported on a genome-wide scale and lacks a conserved mechanism [24]. Consistent with previous publications [17], our “meta” data for genes showed that the H3K4me3 signals, on average, are enriched at the 5′ end of genes (Fig. 5a). Previous studies have focused on the mechanism of this enrichment and found that H3K4me3 depends on the phosphorylation of the C-terminal domain of RNA polymerase II at serine 5 by TFIIH-associated kinase [25]. This phosphorylation signal has a sharp peak at the 5′ region of the gene body [25], which could explain why the H3K4me3 signal is predominantly found at the 5′ end of the gene. Ng et al. [25] proposed that H3K4me3 may provide a molecular memory of recent transcriptional activity. This theory is based on the finding that H3K4me3 persist within the mRNA coding region for a considerable time after transcriptional inactivation and Set1 (yeasthistoneH3-lysine4 (H3-K4)methylase) dissociation from the chromatin [25]. In plants, the flowering of the *Arabidopsis* shoot was studied with a focus on the dynamics of gene expression and H3K4me3 markers, and the results suggested a general congruence between the H3K4me3 dynamics and gene expression changes. However, no precise correlation *r* value has been calculated [26]. Our results in the allotetraploid cotton *G. barbadense* were similar; the H3K4me3 modification represented an active trend for gene expression (Fig. 7).

## Conclusions

In summary, we developed effective CUT&Tag protocols and refined conditions that can be widely used in plants for chromatin profiling. We showed that CUT&Tag outperforms the traditional chromatin profiling method of chromatin immunoprecipitation (ChIP) in allotetraploid cotton plants in terms of operational simplicity and experimental time needed. Most importantly, CUT&Tag needs less starting materials and generates high-resolution signals with low background noise. Our optimized CUT&Tag protocols specifically designed for plant cells had a broad spectrum of for plant epigenetic research.

## Methods

### Plant materials

The allotetraploid cotton cultivar *Gossypium barbadense* (accession H7124) was used in this study. Cotton seedlings were grown in pots at 28 °C in a greenhouse in a 16/8-h light/dark cycle with 60% humidity. Leaf and root samples were collected when the seedlings had two or three true leaves (i.e., from 4-week-old seedlings). Fiber samples were collected from 20 D cotton bolls of H7124.

### Reagents

#### Enzymes

Hyperactive pG-Tn5/pA-Tn5 transposase for CUT&Tag (Vazyme, cat. no. S602/S603); TruePrep Amplify Enzyme (TAE, Vazyme cat. no. TD601).

Note: Check the antibody affinity of the protein A or protein G that is fused with the Tn5. Generally speaking, proteins A and G have broad antibody affinity. However, protein A has a relatively higher affinity to rabbit antibodies and protein G has a relatively higher affinity to mouse antibodies. Select the appropriate transposase products that match your primary antibody.

#### Antibodies

H3K4me3 (Millipore cat. no. 07-473, 1 mg/mL), which is a rabbit polyclonal antibody for detection of histone3 trimethylation at lysine 4; normal rabbit IgG (Millipore cat. no. 12-370, 1 mg/mL), which is used as a control antibody in the CUT&Tag experiment.

#### Chemicals

Tris base; protease inhibitor cocktail (Calbiochem, cat. no. 539133-1SET); Triton X-100; digitonin (~ 50% (TLC), Sigma-Aldrich, cat. no. D141); dimethyl sulfoxide (DMSO); ethylenediaminetetraacetic acid (EDTA); magnesium chloride (MgCl_2_); sodium chloride (NaCl); spermidine; sodium dodecyl sulfate (SDS); bis (trimethylsilyl) acetamide (BSA); phenol:chloroform:isoamyl alcohol (25:24:1,v:v:v); chloroform; 100% ethanol; GlycoBlue Coprecipitant (15 mg/mL, Invitrogen, cat. no. AM9516).

### Equipment

NanoDrop spectrophotometer; centrifuge; Miracloth (Millpore, cat. no. 475855); Eppendorf microcentrifuge tubes.

### Stock solutions

- 1 M Tris pH = 8.0
- 1 M potassium chloride (KCl)
- 1 M magnesium chloride (MgCl_2_)
- 20% Triton X-100
- 0.5 M EDTA (pH = 8.5)
  Note: Making 100 mL of 0.5-M EDTA (pH = 8.5) requires approximately 2 g of sodium hydroxide (NaOH) pellets to adjust the pH.
- 10% SDS
  Note: Do not autoclave; sterilize using a 0.22-micron filter.
- 5 M sodium chloride (NaCl)
- 2.5% digitonin (100 mg digitonin [~ 50% purity] to 2 mL DMSO)
  Note: Sterilize using a 0.22- micron filter.
- 3 M sodium acetate (NaAc)
- Oligos (refer to Additional file 1: Table S1 for sequence information)

### Working solutions

Prepare fresh working solutions; refer to Additional file 1: Table S2 for detailed recipes.

- Annealing buffer for adapters (10 mM Tris pH 8.0, 50 mM NaCl, 1 mM EDTA)
- Nuclear isolation buffer A (10 mM Tris pH 8.0, 10 mM KCl, 0.5 mM spermidine), 50 mL for one sample
- Nuclear isolation buffer B (10 mM Tris pH 8.0, 10 mM KCl, 0.5 mM spermidine, 0.5% Triton X-100, 0.1% cocktail)
- Nuclear wash buffer (10 mM Tris pH 8.0, 150 mM NaCl, 0.5 mM spermidine, protease inhibitor cocktail 0.1%)
- Antibody buffer (50 mM Tris pH 8.0, 1 mM EDTA, 150 mM NaCl, 0.5 mM spermidine, 1 mg/mL BSA, protease inhibitor cocktail 0.1%, 0.05% w/v digitonin)
- Immunoprecipitation (IP) wash buffer (10 mM Tris pH 8.0, 150 mM NaCl, 0.5 mM spermidine, protease inhibitor cocktail 0.1%, 0.05% v/v Tween), 25 mL is enough for eight IP tubes
- Transposase incubation buffer (20 mM Tris pH 8.0, 300 mM NaCl, 0.5 mM spermidine, protease inhibitor cocktail 0.1%, 0.05% w/v digitonin), enough for eight tubes of reaction
- Tagmentation buffer (20 mM Tris pH = 8.0, 300 mM NaCl, 0.5 mM spermidine, protease inhibitor cocktail 0.1%, 10 mM MgCl_2_, 0.05% w/v digitonin) enough for eight tubes of reaction

### Protocol for CUT&Tag assay

#### Making transposase (Day 1)

Make transposase following the manual of hyperactive pG-Tn5/pA-Tn5 transposase for CUT&Tag kit (Vazyme, cat. no. S602/S603).

1. Add **annealing buffer for adapters** to primer A, primer B, and primer C to make a 100-µM stock solution.
2. Set up the following two reactions in two PCR tubes to anneal the adapters: Reaction 1: For a total volume of 20 µL, add 10 µL of 100-µM primer A and 10 µL of 100-µM primer B. Reaction 2: For a total volume of 20 µL, add 10 µL of 100-µM primer A and 10 µL of 100-µM primer C. Anneal the oligos using the program in the PCR machine (heat lid, 75 °C for 15 min, 60 °C for 10 min, 50 °C for 10 min, 40 °C for 10 min, 25 °C for 30 min).
3. Mix the products from Reaction 1 and Reaction 2 at a 1:1 ratio, designated as the adapter mix.
4. Set up the following reaction to generate the transposase: For a total volume of 9.375 μL, add 5 μL of hyperactive pA-Tn5 transposase (500 ng/μL), 0.875 μL of adapter mix, and 3.5 μL of coupling buffer.
  Pipette gently 20 times and mix well, at 30 °C for 1 h. The transposase product is designated as the TTE mix. Store at −20 °C; concentration = 4 pmol/μL.

#### Performing Assay (Days 2 and 3)

##### Day 2 Nuclear preparation

5. Take 1 g of the leaf tissue to be analyzed in the procedure. Grind the leaves in liquid nitrogen to a fine, dry powder.
6. Resuspend the ground and frozen leaf powder (1 g) in a 50-mL tube containing 30 mL of nuclear isolation buffer A (ice cold), and mix immediately with gentle shaking. Filter the solution through two layers of Miracloth, and put the filtered solution in a new ice-cold 50-mL tube. Centrifuge the filtrate for 5 min at 600 × *g* at 4 °C.
  Note: If using a starting material with low input, skip the filter action through the Miracloth step.
7. Remove the supernatant, and add 5 mL of nuclear isolation buffer B (4 °C) to the pellet cells. Transfer the solution immediately to five 1.5-mL tubes (1 mL/each tube; use end-cut tips to transfer). Centrifuge for 3 min at 600 × *g* at 4 °C.
8. For each tube, wash the pellet three times using 1 mL of nuclear wash buffer.
9. For each tube, resuspend the nuclei in 1 mL of antibody buffer. Take 150 μL aliquot of the nuclei suspension using end-cut tips to a 1.5-mL tube for one reaction. An amount of 1 mL of nuclei can be set up for six reactions.
10. Add 1 μL of antibody (anti-H3K4me3 antibody or IgG control antibody) to each reaction (1:50 to 1:100 diluted; the final concentration of antibody is 10–20 μg/mL). Perform immunoprecipitation overnight at 4 °C with gentle shaking.

##### Day 3 Transposase incubation

11. Add 800 μL of IP wash buffer to each reaction. Sit the tubes at room temperature for 5 min, and then centrifuge for 3 min at 300 × *g* at 4 °C to collect the nuclear pellet. Repeat the nuclear pellet washing step for three times.
12. Add 9.375 μL of transposase (generated on Day 1) to 1 mL of transposase incubation buffer, and mix gently.
13. Add 150 μL of transposase from the above step to each reaction. Immunoprecipitate for 1 h at room temperature with gentle shaking.
14. Wash with 800 μL of IP wash buffer, Sit the tubes at room temperature for 5 min, and then centrifuge for 3 min at 300 × *g* at 4 °C to collect the nuclear pellet. Repeat the nuclear pellet washing step for three times.

### Tagmentation

15. Add 300 µL of tagmentation buffer to each reaction, and incubate for 1 h at 37 °C in a water bath.

### DNA extraction

16. Add 10 µL of 0.5-M EDTA and 30 µL of 10% SDS (final concentration 1%) to each reaction to stop the tagmentation.
17. Add 300 µL of DNA extraction buffer as previously reported [27]. Place in a 65 °C water bath for 30 min for nucleic lysis.
18. Add 600 µL phenol:chloroform:isoamyl alcohol to each tube. After shaking, centrifuge the tube for 10 min at 13,000 × *g* at 4 °C to collect the supernatant (~ 600 µL).
19. Add 600 µL chloroform to each tube. After shaking, centrifuge the tube for 10 min at 13,000 × *g* at 4 °C to collect the supernatant (~ 600 µL).
20. Add 1200 µL of 100% ethanol and 2 µL of GlycoBlue Coprecipitant to the supernatant. Store at −20 °C for 1 h, and centrifuge for 10 min at 1,3000 × *g* at 4 °C to collect the DNA.
21. Wash using 75% ethanol.
22. Dissolve the DNA in 24 µL double-distilled water (ddH_2_O).

### Library construction

23. Set up the PCR reaction as follows using the TruePrep Amplify Enzyme (TAE, Vazyme): For a total volume of 50 µL, add 24 µL of DNA, 11 µL of ddH_2_O, 10 µL of 5 × TAE buffer, 2 µL of 10-µM P5 primer X, 2 µL of 10-µM P7 primer X, and 2 µL of TAE. Mix gently and spin briefly.
  Note: For the number of reactions and selection criteria for P5 primer X and P7 primer X, refer to the Index Adapter Pooling Guide for Illumina (e.g., TruePrep® Index Kit V2 for Illumina, Vazyme TD202); for a detailed sequence of P5 primer X and P7 primer X and the Index Adapter Pooling Guide strategy in this study, refer to Additional file 1: Tables S1 and S3.
24. Set up the PCR program: 72 °C for 3 min, 98 °C for 30 s; then 16–18 cycles of 98 °C for 30 s, 60 °C for 30 s, and 72 °C for 30 s, followed by 72 °C for 5 min.
  Note: Overamplification of the library will lead to high levels of PCR duplicates in NGS. For histone H3K4me3 modification, 16–18 cycles are recommended when using the 100-µL nuclei described above in the protocol (equals approximately 1 µg of chromatin). Generally, using 20 PCR cycles is commended when using starting nuclei of less than 1 k; 17–18 cycles for 1 k to 1 week, and 15–17 cycles for 1–10 week. The criteria for PCR cycle selection are starting with low numbers of cycles and increasing the numbers if needed. In this way the library has enough enrichment of fragments at low levels of PCR duplicates to achieve high “complexity” for NGS.

### PCR product purification

25. Purify the PCR products using a commercial column or beads.
26. Load 2 µL of the purification product on 2% agarose gel for electrophoresis to detect the fragment concentration and distribution.
27. Use Qubit fluorometric quantitation to detect the library concentration and quality.

### Next-generation sequencing and bioinformatic analysis

28. Perform paired-end Illumina sequencing on the bar-coded libraries using an Illumina HiSeq 2500 or another massively parallel DNA sequencer, following the manufacturer’s instructions. Obtain a 6- to 7-G raw base data.
29. Fastp v 0.20.1 [28] is used to remove adapter and low-quality reads. Align paired-end reads using Hisat2 v 2.2.0 [29] with the following parameters: –no-spliced-alignment–no-mixed–no-discordant–phred33 -I 10 -X 700. Unique aligned reads are extracted using perl script: cat aligned.sam | perl -ne “print if /^@|NH:i:1\b/”. Duplicated reads are removed using Picard v 2.22.8 (Picard Toolkit 2019, Broad Institute, GitHub Repository, https://broadinstitute.github.io/picard/) with this parameter: REMOVE_DUPLICATES = true. Peak calling uses macs2 v 2.1.3.3 [30] with these parameters: macs2 callpeak -t input_file -p 1e-5 -f BAM -keep-dup all -n out_name. Scatterplots, correlation plots, and heatmaps are displayed using deepTools v 3.1.3 [31]. Annotation of peaks is performed using an R/Bioconductor package ChIPseeker [32].

## Supplementary information

**Additional file 1: Figure S1.** Qubit fluorometric quantitation of DNA libraries.** Figure S2.** Correlation analysis of H3K4me3 peak intensities and gene expression.** Figure S3.** Heatmap of H3K4me3 signals near PCGs with TPM values in descending order.** Table S1.** Oligos used in this study.** Table S2.** Recipes for working solutions.** Table S3.** Index Adapter Pooling Guide strategy used in this study.

## Data Availability

Novel data generated in this study, including raw data of CUT&Tag, CHIP, and transcriptome sequencing, have been deposited in the National Center for Biotechnology Information under the accession number PRJNA641980.

## Abbreviations

ATAC-seq: Assay for transposase-accessible chromatin with high throughput sequencing
ChEC-seq: Chromatin endogenous cleavage sequencing
ChIP: Chromatin immunoprecipitation
CUT&RUN: Cleavage under targets and release using nuclease
CUT&Tag: Cleavage under targets and tagmentation
DamID: DNA adenine methyltransferase identification
FAIRE-seq: Formaldehyde-assisted isolation of regulatory elements sequencing
MNase-seq: Micrococcal nuclease sequencing
PCR: Polymerase chain reaction
PCGs: Protein-coding genes
Sono-seq: Sonication of cross-linked chromatin sequencing
TPM: Transcripts per kilobase of exon model per million mapped reads
CPM: Count per million mapped reads

## References

[1] Deng X, Song X, Wei L, Liu C, Cao X (2016). Epigenetic regulation and epigenomic landscape in rice. Natl Sci Rev.

[2] Kharchenko PV, Tolstorukov MY, Park PJ (2008). Design and analysis of ChIP-seq experiments for DNA-binding proteins. Nat Biotechnol.

[3] Kaya-Okur HS, Wu SJ, Codomo CA, Pledger ES, Bryson TD, Henikoff JG, Ahmad K, Henikoff S (2019). CUT&Tag for efficient epigenomic profiling of small samples and single cells. Nat Commun.

[4] Crawford GE, Holt IE, Whittle J, Webb BD, Tai D, Davis S, Margulies EH, Chen Y, Bernat JA, Ginsburg D (2006). Genome-wide mapping of DNase hypersensitive sites using massively parallel signature sequencing (MPSS). Genome Res.

[5] Giresi PG, Kim J, McDaniell RM, Iyer VR, Lieb JD (2007). FAIRE (Formaldehyde-Assisted Isolation of Regulatory Elements) isolates active regulatory elements from human chromatin. Genome Res.

[6] Auerbach RK, Euskirchen G, Rozowsky J, Lamarre-Vincent N, Moqtaderi Z, Lefrançois P, Struhl K, Gerstein M, Snyder M (2009). Mapping accessible chromatin regions using Sono-Seq. Proc Natl Acad Sci.

[7] Kent NA, Adams S, Moorhouse A, Paszkiewicz K (2011). Chromatin particle spectrum analysis: a method for comparative chromatin structure analysis using paired-end mode next-generation DNA sequencing. Nucleic Acids Res.

[8] Buenrostro JD, Giresi PG, Zaba LC, Chang HY, Greenleaf WJ (2013). Transposition of native chromatin for fast and sensitive epigenomic profiling of open chromatin, DNA-binding proteins and nucleosome position. Nat Methods.

[9] van Steensel B, Delrow J, Henikoff S (2001). Chromatin profiling using targeted DNA adenine methyltransferase. Nat Genet.

[10] Schmid M, Durussel T, Laemmli UK (2004). ChIC and ChEC; genomic mapping of chromatin proteins. Mol Cell.

[11] Skene PJ, Henikoff JG, Henikoff S (2018). Targeted in situ genome-wide profiling with high efficiency for low cell numbers. Nat Protoc.

[12] Zheng XY, Gehring M (2019). Low-input chromatin profiling in Arabidopsis endosperm using CUT&RUN. Plant Reprod.

[13] Zhang T, Hu Y, Jiang W, Fang L, Guan X, Chen J, Zhang J, Saski CA, Scheffler BE, Stelly DM (2015). Sequencing of allotetraploid cotton (Gossypium hirsutum L. acc. TM-1) provides a resource for fiber improvement. Nat Biotechnol.

[14] Hu Y, Chen J, Fang L, Zhang Z, Ma W, Niu Y, Ju L, Deng J, Zhao T, Lian J (2019). Gossypium barbadense and Gossypium hirsutum genomes provide insights into the origin and evolution of allotetraploid cotton. Nat Genet.

[15] Landt SG, Marinov GK, Kundaje A, Kheradpour P, Pauli F, Batzoglou S, Bernstein BE, Bickel PJ, Brown J, Cayting P (2012). ChIP-seq guidelines and practices of the ENCODE and modENCODE consortia. Genome Res.

[16] Barski A, Cuddapah S, Cui K, Roh TY, Schones DE, Wang Z, Wei G, Chepelev I, Zhao K (2007). High-resolution profiling of histone methylations in the human genome. Cell.

[17] Pokholok DK, Harbison CT, Levine S, Cole M, Hannett NM, Lee TI, Bell GW, Walker K, Rolfe PA, Herbolsheimer E (2005). Genome-wide map of nucleosome acetylation and methylation in yeast. Cell.

[18] Robinson JT, Thorvaldsdóttir H, Winckler W, Guttman M, Lander ES, Getz G, Mesirov JP (2011). Integrative genomics viewer. Nat Biotechnol.

[19] Li X, Wang X, He K, Ma Y, Su N, He H, Stolc V, Tongprasit W, Jin W, Jiang J (2008). High-resolution mapping of epigenetic modifications of the rice genome uncovers interplay between DNA methylation, histone methylation, and gene expression. Plant Cell.

[20] Wang X, Elling AA, Li X, Li N, Peng Z, He G, Sun H, Qi Y, Liu XS, Deng XW (2009). Genome-wide and organ-specific landscapes of epigenetic modifications and their relationships to mRNA and small RNA transcriptomes in maize. Plant Cell.

[21] Chen K, Chen Z, Wu D, Zhang L, Lin X, Su J, Rodriguez B, Xi Y, Xia Z, Chen X (2015). Broad H3K4me3 is associated with increased transcription elongation and enhancer activity at tumor-suppressor genes. Nat Genet.

[22] Haring M, Offermann S, Danker T, Horst I, Peterhansel C, Stam M (2007). Chromatin immunoprecipitation: optimization, quantitative analysis and data normalization. Plant Methods.

[23] Santos-Rosa H, Schneider R, Bannister AJ, Sherriff J, Bernstein BE, Emre NCT, Schreiber SL, Mellor J, Kouzarides T (2002). Active genes are tri-methylated at K4 of histone H3. Nature.

[24] Howe FS, Fischl H, Murray SC, Mellor J (2017). Is H3K4me3 instructive for transcription activation. BioEssays.

[25] Ng HH, Robert F, Young RA, Struhl K (2003). Targeted recruitment of Set1 histone methylase by elongating pol II provides a localized mark and memory of recent transcriptional activity. Mol Cell.

[26] You Y, Sawikowska A, Neumann M, Pose D, Capovilla G, Langenecker T, Neher RA, Krajewski P, Schmid M (2017). Temporal dynamics of gene expression and histone marks at the Arabidopsis shoot meristem during flowering. Nat Commun.

[27] Paterson AH, Brubaker CL, Wendel JF (1993). A rapid method for extraction of cotton (Gossypium spp.) genomic DNA suitable for RFLP or PCR analysis. Plant Mol Biol Rep.

[28] Chen S, Zhou Y, Chen Y, Gu J (2018). fastp: an ultra-fast all-in-one FASTQ preprocessor. Bioinformatics.

[29] Kim D, Langmead B, Salzberg S (2015). HISAT: A fast spliced aligner with low memory requirements. Nat Methods.

[30] Zhang Y, Liu T, Meyer CA, Eeckhoute J, Johnson DS, Bernstein BE, Nusbaum C, Myers RM, Brown M, Li W (2008). Model-based analysis of ChIP-Seq (MACS). Genome Biol.

[31] Ramirez F, Dundar F, Diehl S, Gruning BA, Manke T (2014). deepTools: a flexible platform for exploring deep-sequencing data. Nucleic Acids Res.

[32] Yu G, Wang L, He Q (2015). ChIPseeker: an R/Bioconductor package for ChIP peak annotation, comparison and visualization. Bioinformatics.

