# Supplementary material for: Efficient chromatin profiling of H3K4me3 modification in cotton using CUT&Tag
Source: Plant Methods. 2020 Aug 31;16:120. doi: 10.1186/s13007-020-00664-8 (PMC7460760; doi:10.1186/s13007-020-00664-8)
Supplement: Supplementary file 1 — Additional file 1: Figure S1. Qubit fluorometric quantitation of DNA libraries. Figure S2. Correlation analysis of H3K4me3 peak intensities and gene expression. Figure S3. Heatmap of H3K4me3 signals near PCGs with TPM values in descending order. Table S1. Oligos used in this study. Table S2. Recipes for working solutions. Table S3. Index Adapter Pooling Guide strategy used in this study. [file 13007_2020_664_MOESM1_ESM.docx]

**Figure S1**


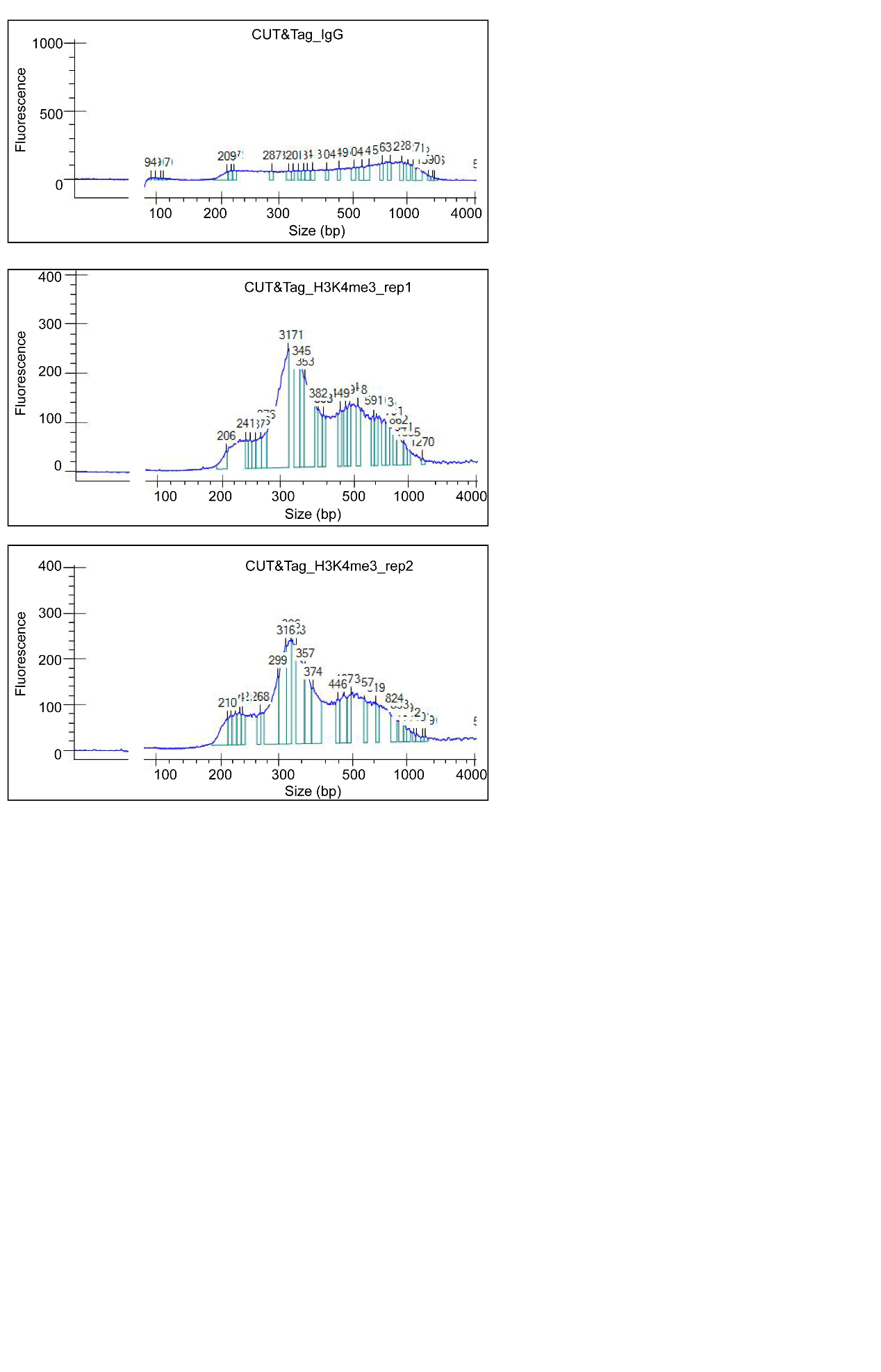


**Figure S1** Qubit fluorometric quantitation of DNA libraries.

**Figure S2**


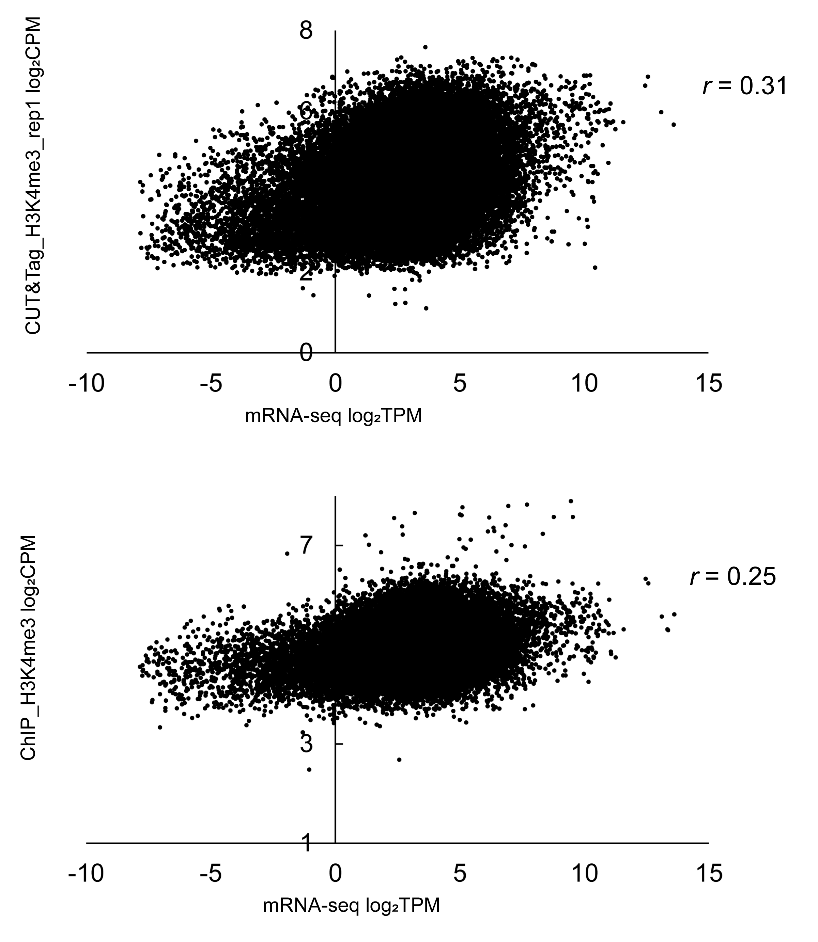


**Figure S2** Correlation analysis of H3K4me3 peaks intensities and gene expression. Signals of the peaks was normalized by log2 value of CPM. Gene expression level was normalized by log2 value of TPM.

**Figure S3**

**
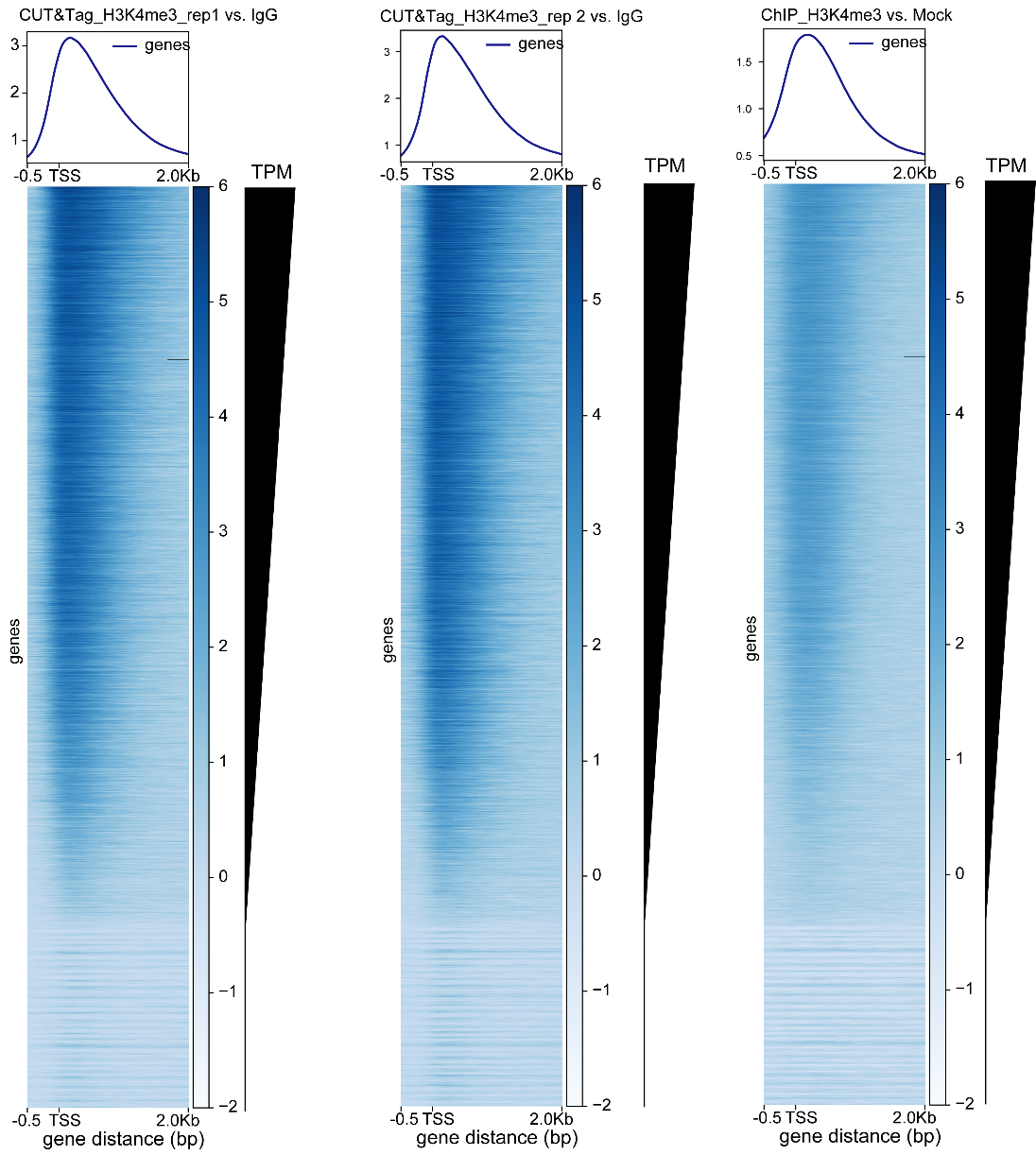
**

**Figure S3** Heatmap of H3K4me3 signals near PCGs with TPM value sorting in descending order.

**Table S1 Oligos used in this study.**

| **Primer** | **Sequences** | **Length** | **Purification Method** | **Usage** |
| --- | --- | --- | --- | --- |
| Primer A | 5'-phos-CTGTCTCTTATACACATCT-NH2 -3' (5'-Phosphate, 3'-AminolinkerC7) | 34 | HPLC |  |
| Primer B | 5′-TCGTCGGCAGCGTCAGATGTGTATAAGAGACAG-3′ | 39 | DSL |  |
| Primer C | 5′-GTCTCGTGGGCTCGGAGATGTGTATAAGAGACAG-3′ | 40 | DSL |  |
| Primer 1 | 5′-AATGATACGGCGACCACCGA-3′ | 26 | DSL |  |
| Primer 2 | 5′-CAAGCAGAAGACGGCATACGA-3′ | 27 | DSL |  |
| N501 | 5′-AATGATACGGCGACCACCGAGATCTACACTAGATCGCTCGTCGGCAGCGTC-3′ | 57 | DSL |  |
| N502 | 5′-AATGATACGGCGACCACCGAGATCTACACCTCTCTATTCGTCGGCAGCGTC-3′ | 57 | DSL |  |
| N701 | 5′-CAAGCAGAAGACGGCATACGAGATTAAGGCGAGTCTCGTGGGCTCGG-3′ | 53 | DSL |  |
| N702 | 5′-CAAGCAGAAGACGGCATACGAGATCGTACTAGGTCTCGTGGGCTCGG-3′ | 53 | DSL |  |
| N703 | 5′-CAAGCAGAAGACGGCATACGAGATAGGCAGAAGTCTCGTGGGCTCGG-3′ | 53 | DSL |  |
| N704 | 5′-CAAGCAGAAGACGGCATACGAGATTCCTGAGCGTCTCGTGGGCTCGG-3′ | 53 | DSL |  |
| N705 | 5′-CAAGCAGAAGACGGCATACGAGATGGACTCCTGTCTCGTGGGCTCGG-3′ | 53 | DSL |  |
| N706 | 5′-CAAGCAGAAGACGGCATACGAGATTAGGCATGGTCTCGTGGGCTCGG-3′ | 53 | DSL |  |

Index sequences were indicated as red.

**Table S2** **Recipes for working solutions.**

| **Annealing buffer for adapters** | 10 mM Tris pH 8.0, 50 mM NaCl, 1 mM EDTA |
| --- | --- |
| Stock solutions | Volume |
| 1 M Tris pH 8.0 | 0.1 mL |
| 5 M NaCl | 0.1 mL |
| 0.5 M EDTA | 20 µL |
| Total | 10 mL |
|  |  |
| **Nuclear Isolation buffer A** |  |
| Stock solutions | Volume |
| 1 M Tris pH 8.0 | 0.5 mL |
| 1 M KCl | 0.5 mL |
| 2 M Spermidine | 12.5 µL |
| ddH_2_O | 49 mL |
| Total | 50 mL |
|  |  |
| **Nuclear Isolation buffer B** |  |
| Stock solutions | Volume |
| Isolation buffer A | 5 mL |
| 20% Triton ×-100 | 125 µL |
| Proteinase inhibitors cocktail | 5 µL |
| Total | 5 mL |
|  |  |
| **Nuclear Wash buffer** |  |
| Stock solutions | Volume |
| 1 M Tris pH 8.0 | 0.5 mL |
| 5 M NaCl | 1.5 mL |
| 2 M Spermidine | 12.5 µL |
| Proteinase inhibitors cocktail | 50 µL |
| ddH_2_O | 48 mL |
| Total | 50 mL |
|  |  |
| **Antibody buffer** |  |
| Stock solutions | Volume |
| 1 M Tris pH 8.0 | 0.25 mL |
| 0.5 M EDTA | 10 µL |
| 5 M NaCl | 150 µL |
| 2 M Spermidine | 1.25 µL |
| Proteinase inhibitors cocktail | 5 µL |
| 10 mg/ml BSA | 0.5 mL |
| 2.5% w/v Digitonin | 100 µL |
| ddH_2_O | 4 mL |
| Total | 5 mL |
|  |  |
| **IP Wash buffer** |  |
| Stock solutions | Volume |
| Nuclear wash buffer | 25 mL |
| 20% v/v Tween | 6.25 µL |
| Total | 25.00625 mL |
|  |  |
| **Transposase incubation buffer** |  |
| Stock solutions | Volume |
| IP Wash buffer | 1 mL |
| 5 M NaCl | 30 µL |
| 2.5% w/v Digitonin | 20 µL |
| Total | 1.05 mL |
|  |  |
| **Tagmentation buffer** |  |
| Stock solutions | Volume |
| IP Wash buffer | 3 mL |
| 5 M NaCl | 90 µL |
| 1 M MgCl2 | 30 µL |
| 2.5% w/v Digitonin | 60 µL |
| Total | 3.18 mL |

**Table S3 Index Adaptor pooling strategy in this study.**

| **Sample name** | P7 primer X | P5 primer X | **Index 1 (i7)** | **Index 2 (i5)** |
| --- | --- | --- | --- | --- |
| Cuttag_H3K4me3_rep1 | N704 in Table S1 | N501 | TCCTGAGC | TAGATCGC |
| Cuttag_H3K4me3_rep2 | N705 | N501 | GGACTCCT | TAGATCGC |
| Cuttag_IgG | N701 | N501 | TAAGGCGA | TAGATCGC |
